# Less (Transfusion) Is More—Enhancing Recovery through Implementation of Patient Blood Management in Cardiac Surgery: A Retrospective, Single-Centre Study of 1174 Patients

**DOI:** 10.3390/jcdd10070266

**Published:** 2023-06-22

**Authors:** Mihai Ștefan, Dana Tomescu, Cornelia Predoi, Raluca Goicea, Mihai Perescu, Mihai Popescu, Dan Dorobanțu, Gabriela Droc, Ștefan Andrei, Ovidiu Știru, Șerban-Ion Bubenek Turconi, Daniela Filipescu

**Affiliations:** 1 2nd Department of Anaesthesiology and Intensive Care, “Prof Dr CC Iliescu” Emergency Institute for Cardiovascular Diseases, 022322 Bucharest, Romania; cornelia-elena.florescu@drd.umfcd.ro (C.P.);; 2 Discipline of Anaesthesiology and Intensive Care, “Carol Davila” University of Medicine and Pharmacy, 419291 Bucharest, Romania; 3 3rd Department of Anaesthesiology and Intensive Care, Fundeni Clinical Institute, 022328 Bucharest, Romania; 4 Children’s Health and Exercise Research Center, University of Exeter, Exeter EX4 4QJ, UK; 5 Faculty of Health Sciences, University of Bristol, Bristol BS8 1TH, UK; 6 Congenital Heart Unit, Bristol Royal Hospital for Children and Heart Institute, Bristol BS2 8ED, UK; 7 1st Department of Anaesthesiology and Intensive Care, Fundeni Clinical Institute, 022322 Bucharest, Romania; 8 Department of Anaesthesiology and Critical Care Medicine, Dijon University Medical Centre, 21000 Dijon, France; 9 Department of Cardiovascular Surgery, “Prof Dr CC Iliescu” Emergency Institute for Cardiovascular Diseases, 419291 Bucharest, Romania; 10 Discipline of Cardiovascular Surgery, “Carol Davila” University of Medicine and Pharmacy, 419291 Bucharest, Romania; 11 1st Department of Anaesthesiology and Intensive Care, “Prof Dr CC Iliescu” Emergency Institute for Cardiovascular Diseases, 022322 Bucharest, Romania

**Keywords:** transfusion, anaemia, cardiac surgery, patient blood management

## Abstract

**Introduction:** The implementation of Patient Blood Management (PBM) in cardiac surgery has been shown to be effective in reducing blood transfusions and associated complications, as well as improving patient outcomes. Despite the potential benefits of PBM in cardiac surgery, there are several barriers to its successful implementation. **Objectives:** The main objectives of this study were to ascertain the impact of the national Romanian PBM recommendations on allogeneic blood product transfusion in cardiac surgery and identify predictors of perioperative packed red blood cell transfusion. **Methods:** As part of the Romanian national pilot programme of PBM, we performed a single-centre, retrospective study in a tertiary centre of cardiovascular surgery, including patients from two time periods, before and after the implementation of the national recommendations. Using coarsened exact matching, from a total of 1174 patients, 157 patients from the before group were matched to 169 patients in the after group. Finally, we built a multivariate regression model from the entire cohort to analyse independent predictors of PRBC transfusion in the perioperative period. **Results:** Although there was a trend towards a lower proportion of patients requiring PRBC transfusion in the “after” group compared to the “before” group (44.9%vs. 50.3%), it was not statistically significant. There was a significant difference between the “after” group and the “before” group in terms of fresh-frozen plasma (FFP) transfusion rates, with a lower percentage of patients requiring FFP transfusion in the “after” group compared to “before” (14.2%, vs. 22.9%, *p* = 0.04). This difference was also seen in the total perioperative FFP transfusion (mean transfusion 0.7 units in the “before” group, SD 1.73 vs. 0.38 units in the “after” group, SD 1.05, *p* = 0.04). In the multivariate regression analysis, age > 64 years (OR 1.652, 95% CI 1.17–2.331, *p* = 0.004), female sex (OR 2.404, 95% CI 1.655–3.492, *p* < 0.001), surgery time (OR 1.295, 95% CI 1.126–1.488, *p* < 0.001), Hb < 13 g/dl (OR 3.611, 95% CI 2.528–5.158, *p* < 0.001), re-exploration for bleeding (OR 3.988, 95% CI 1.248–12.738, *p* = 0.020), viscoelastic test use (OR 2.18, 95% CI 1.34–3.544, *p* < 0.001), FFP transfusion (OR 4.023, 95% CI 2.426–6.671, *p* < 0.001), and use of a standardized pretransfusion checklist (OR 8.875, 95% CI 5.496–14.332, *p* < 0.001) remained significantly associated with PRBC transfusion. The use of a preoperative standardized haemostasis questionnaire was independently associated with a decreased risk of perioperative PRBC transfusion (0.565, 95% CI 0.371–0.861, *p* = 0.008). **Conclusions:** Implementation of national PBM recommendations led to a reduction in FFP transfusion in a cardiac surgery centre. The use of a preoperative standardized haemostasis questionnaire is an independent predictor of a lower risk for PRBC transfusion in this setting.

## 1. Introduction

Cardiac surgery is a complex and high-risk procedure that often requires the use of blood products to replace blood loss and support the patient’s cardiovascular system [1,2]. However, blood transfusions are not without risks, including transfusion reactions, infections, and immunological complications [3,4]. Additionally, transfusions can be costly and may not always be necessary [5,6].

PBM is a methodical and evidence-based approach that prioritizes the patient and aims to enhance their outcomes by safeguarding and utilizing their own blood. This approach also emphasizes patient safety and empowerment [7]. PBM is proactive, multidisciplinary, and multimodal, and it includes anaemia detection and treatment, haemostasis optimisation, blood loss minimisation, the rational use of blood products, and the optimisation of anaemia tolerance to strengthen and to preserve patients’ own blood mass, to enable the safe handling of donor blood, and to improve the patient’s outcome/prognosis [8,9].

The implementation of PBM in cardiac surgery has been shown to be effective in reducing blood transfusions and associated complications, as well as improving patient outcomes such as mortality, morbidity, and length of hospital stay [10]. However, the implementation of PBM requires a multidisciplinary approach and significant changes in clinical practice, which can be challenging.

Despite the potential benefits of implementing PBM in cardiac surgery, there are several barriers to its successful implementation [11,12]. Some of the most common include a lack of knowledge and awareness among healthcare providers, resistance to change, inadequate resources, and inadequate communication and collaboration among different healthcare disciplines [13]. In addition, there may be cultural and institutional barriers, as well as differences in local landscape, that can make it difficult to implement PBM programs and lead to significant variability [14,15]. Overcoming these barriers requires a concerted effort from all stakeholders, including hospital administrators, healthcare providers, and patients [16]. By addressing these challenges, PBM can be successfully implemented in cardiac surgery, leading to improved patient outcomes and reduced healthcare costs [17].

In Romania, a multidisciplinary initiative group endorsed by the Ministry of Health has developed national recommendations for PBM, aimed at improving patient outcomes and reducing the use of allogeneic blood transfusions, which are a precious but insufficient resource in the elective surgical setting [18,19]. The recommendations cover a range of areas, including the preoperative optimization of haemoglobin (Hb) levels and haemostasis, intraoperative haemostasis management and protocols, and postoperative monitoring and management of anaemia. They also emphasize the importance of individualizing transfusion decisions based on patient characteristics and clinical factors, rather than relying on fixed transfusion thresholds. The implementation of these recommendations has the potential to reduce the risks associated with blood transfusions, such as transfusion reactions and infections, while improving patient outcomes and optimizing the use of healthcare resources. Previous papers have been published as part of the programme [20,21].

The main objectives of this study were to ascertain the impact of the national PBM recommendations on allogeneic blood product transfusion and identify predictors of perioperative packed red blood cell (PRBC) transfusion in a tertiary care cardiac surgery academic centre.

## 2. Materials and Methods

### 2.1. Patient Population and Data Collection

As part of the Romanian national pilot programme of PBM [19], we performed a single-centre, retrospective study in a tertiary centre of cardiovascular surgery. Data were recorded using a pre-approved protocol, available in Supplementary Table S1.

We included consecutive patients who underwent non-emergent cardiac surgery with cardio-pulmonary bypass (CPB) during two distinct periods: 1 January 2017–30 June 2017 and 1 July 2018–31 December 2018, referred to as the “before” group, and 1 January 2020–31 December 2020, referred to as the “after” group, following the implementation of the Romanian PBM recommendations. These periods of data collection were selected as representing the periods before publishing the recommendations, right at the beginning of the implementation and one year after, respectively. Patients who underwent emergency surgery and those for whom research information could not be adequately collected (incomplete observation sheets) were excluded from the study.

The data were recorded according to the directives of the Order of the Minister of Health no. 1251 of 2018 and their statistical processing for publication was approved by the ethics and study approval committee of the Emergency Institute for Cardiovascular Diseases “Prof. Dr. C. C. Iliescu”, Bucharest, nr. 15324/ 03.06.2021.

All data were recorded retrospectively, based on written observation sheets and electronic logs.

### 2.2. Clinical Management

The surgical procedure was performed under general anaesthesia with intravenous (iv) induction and volatile-based maintenance, utilizing sevoflurane. During CPB, total iv anaesthesia was administered using either propofol or midazolam, and analgesia was achieved through a fentanyl infusion. The CPB was conducted using crystalloid priming and cardioplegia, which involved either a 4:1 mix of blood and crystalloid or Custodiol solution, based on the surgeon’s preference and the anticipated surgery duration. Heparin was administered before bypass at a dose of 350 units per kilogram of body weight and supplemented, if necessary, to achieve an activated clotting time above 480 s, and protamine was used to antagonize heparin at a ratio of 0.8 of the initial heparin doses. Cell saver and intraoperative hemofiltration were used only in complex cases, at the attending anaesthesiologist’s discretion. Point-of-care viscoelastic haemostasis monitoring was available and primarily used in high-risk patients, utilizing rotation thromboelastometry (ROTEM) and an institutional bleeding management algorithm that included allogeneic blood products and factor concentrates. All patients received tranexamic acid prophylactically. The decision to administer PRBC transfusions was based on a restrictive-oriented approach, with Hb triggers of 7 g/dL during CPB and 8 g/dL during the rest of the perioperative period, while considering other factors such as patient characteristics, (mixed venous blood saturation of oxygen) SvO2, and lactate level. The recorded outcomes included the duration of surgery; hospital stay; surgical re-exploration due to bleeding; PRBC transfusion pre-, intra-, and postoperatively; the use of ROTEM and cell saver; and FFP and platelet (PLT) transfusion. Haemoglobin (Hb) values were recorded at four distinct time points: Hb1—at hospital admission, Hb 2—last Hb recorded preoperatively, Hb3—first Hb recorded postoperatively, and Hb4—at hospital discharge. Anaemia was defined as an Hb value < 13 g/dl, regardless of sex, for the analysis.

### 2.3. Statistical Analysis

Data analysis involved the use of Microsoft Excel^®^ (Microsoft Corporation, Redmond, WA, USA), STATA^®^ (StataCorp. 2021. Stata Statistical Software: Release 17. StataCorp LLC, College Station, TX, USA College Station, TX, USA), and Wizard 2 (Wizard–Statistics & Analysis^®^, Raipur, Chattisgarh, India). A *p*-value < 0.05 was considered statistically significant a priori.

Quantitative data were expressed as medians with [25–75%] interquartile ranges (IQR). Normal distribution for continuous variables was evaluated by histograms and the Shapiro–Wilk test. Student’s t-test or Mann–Whitney U-test were used as appropriate for comparisons of continuous variables. Qualitative data were expressed as numbers (percentages). Chi-square or Fisher’s exact test was used to compare categorical variables, as appropriate.

To determine the impact of the adoption of the PBM recommendations on intrahospital transfusion, we performed a coarsened exact matching between patients in the “before” group and those in the “after” group, using age, sex, hospital length of stay (LOS), ICU LOS, the type of surgery, surgery time, preoperative anaemia, preoperative Hb, and the use of ROTEM and cell saver as co-variates and compared the selected cohorts with appropriate statistical tests, as above.

To investigate the association between perioperative variables and transfusion risk, we built a multivariable logistic regression model, using the perioperative transfusion requirement as the dependent variable, and predictors selected by univariate analysis with a *p*-value less than 0.05.

## 3. Results

**Figure 1:**
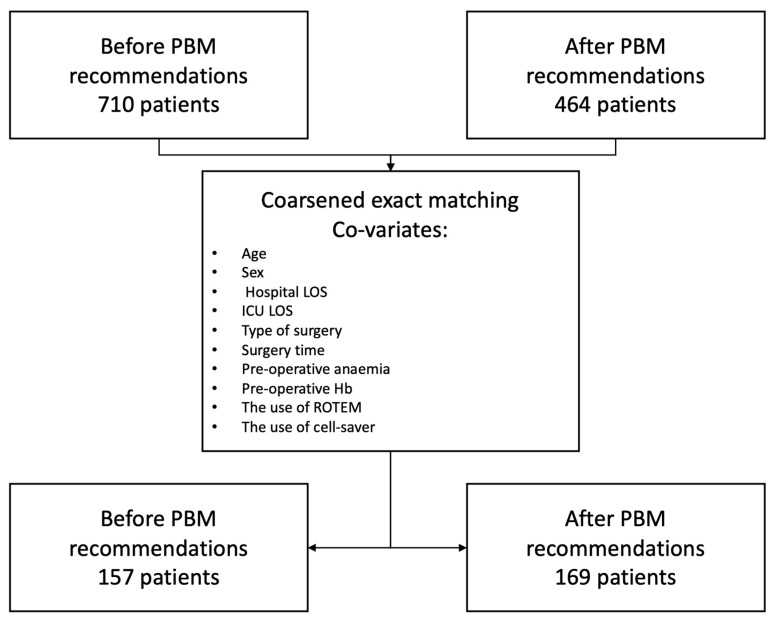
Study flowchart.

This is a before and after study in a non-emergent cardiac surgery population that compared two cohorts, one before the implementation of the PBM national guidelines and the other after. The cohorts have been matched using coarsened exact matching. The study flowchart is available in Figure 1. In total, 710 patients were included in the before group and 464 in the after group. After performing the coarsened exact matching, 157 patients from the before group were matched to 169 patients in the after group.

### Demographic Characteristics and Outcomes

Before matching, there were 710 patients in the “before” group and 464 patients in the “after” group, who had a median age of 64 years (IQR 58–69 vs. 56–69, *p* = 0.643) and were predominantly male (66.1% vs. 65.9%, *p* = 0.970).

Table 1 shows the types of surgeries performed in each time period.

The perioperative co-variates used in the model are available in Table 2. The only statistical differences found between the groups after matching are lower ICU LOS in the “before” group compared to “after” (2 days, IQR 2–3 vs. 3 days, IQR 3–3), and a higher Hb 2 in the “before” group compared to “after” (13.6 g/d, IQR 12.6–14.5, vs. 13.2, IQR 12.1–14.2). There were no significant differences between groups in regard to age, sex, hospital LOS, surgery time, prevalence of preoperative anaemia, Hb1, or the use of ROTEM or cell saver.

Perioperative transfusion outcomes, as well as the implementation of specific guideline measures, such as the standardized preoperative haemostasis questionnaire (Supplementary Figure S1) and the standardized pretransfusion checklist (Supplementary Figure S2), are presented in Table 3.

Although there was a trend towards a lower proportion of patients requiring PRBC transfusion in the “after” group (44.9% vs. 50.3%), it was not statistically significant. Similarly, there was no significant difference between the groups in terms of perioperative PRBC units transfused (1 vs. 0 units per patient, *p* = 0.424). The findings reveal that in the “after” group, there was a decrease in median Hb levels compared to the “before” group, both at point 3–10.1 g/dl (IQR 9.1–11.1) vs. 9.4 (IQR 8.7–10.1) and at point 4–9.8 g/dl (IQR 9.2–10.7) vs. 9.3 g/dl (IQR 8.5–9.9).

There was a significant difference between the “after” group and the “before” group in terms of FFP transfusion rates, with a lower percentage of patients requiring FFP transfusion in the “after” group (14.2%, vs. 22.9%, *p* = 0.04). This difference was also seen in the total number of perioperative FFP units transfused (mean transfusion 0.7 units, SD 1.73 vs. 0.38 units, SD 1.05, *p* = 0.04) and intraoperative FFP transfusion (mean transfusion 0.59 units, SD 1.31, vs. 0.23 units, SD 0.79, *p* = 0.003).

The interval between the preoperative anaesthetic consultation and surgery was statistically different between the groups, but the clinical significance was negligible (median 1 day in both groups, *p* = 0.005, mean 1.3 days, SD 1.4 vs. 0.9 days, SD 0.7, *p* = 0.002).

There was an increase in the utilization of the standardized haemostatic questionnaire, with 75.7% of patients in the “after” group compared to 12.1% of patients in the “before” group (*p* < 0.001).

The standardised pretransfusion checklist was only used in the “after” period.

PLT transfusion rate was not statistically different between the two groups, 6.3% vs. 3.5%, *p* = 0.239.

In the univariate analysis (Table 4), we selected age >64 years (OR 2.31, 95% CI 1.825–2.922, *p* < 0.001), female sex (OR 2.645, 95% CI 2.048–3.415, *p* < 0.001), hospital LOS (OR 1.037, 95% CI 1.021–1.054, *p* < 0.001), ICU LOS (OR 1.168, 95% CI 1.097–1.243, *p* < 0.001), surgery time (OR 1.348, 95% CI 1.219–1.489, *p* < 0.001), Hb < 13 g/dl (OR 4.315, 95% CI 3.34–5.576, *p* < 0.001), re-exploration for bleeding (OR 6.879, 95% CI 3.717–12.733, *p* < 0.001), ROTEM use (OR 4.401, 95% CI 3.053–6.344, *p* < 0.001), FFP transfusion (OR 7.898, 95% CI 5.601–11.135, *p* < 0.001), PLT transfusion (OR 3.32, 95% CI 2.059–5.354, *p* < 0.001), and use of a standardized pretransfusion checklist (OR 8.151, 95% CI 5.73–11.596, *p* < 0.001), which were significantly associated with PRBC transfusion, while the use of a standardized haemostasis questionnaire was inversely associated with PRBC transfusion (OR 0.766, 95% CI 0.6–0.976, *p* = 0.031).

Using these variables as predictors and perioperative transfusion as the dependent variable, we built a multivariate model, and logistic regression analysis showed that age > 64 years (OR 1.652, 95% CI 1.17–2.331, *p* = 0.004), female sex (OR 2.404, 95% CI 1.655–3.492, *p* < 0.001), surgery time (OR 1.295, 95% CI 1.126–1.488, *p* < 0.001), Hb < 13 g/dl (OR 3.611, 95% CI 2.528–5.158, *p* < 0.001), re-exploration for bleeding (OR 3.988, 95% CI 1.248–12.738, *p* = 0.020), ROTEM use (OR 2.18, 95% CI 1.34–3.544, *p* < 0.001), FFP transfusion (OR 4.023, 95% CI 2.426–6.671, *p* < 0.001), and standardized pretransfusion checklist use (OR 8.875, 95% CI 5.496–14.332, *p* < 0.001) remained significantly associated with PRBC transfusion. The use of a preoperative standardized haemostasis questionnaire was independently associated with a decreased risk of perioperative PRBC transfusion (0.565, 95% CI 0.371–0.861, *p* = 0.008).

## 4. Discussion

In this before and after retrospective study, we showed that the implementation of the PBM national recommendations in a cardiac surgery population led to a decrease in FFP transfusion rates and an increase in the utilization of the standardized haemostatic questionnaire, with no significant difference in perioperative PRBC and PLT transfusion rates.

There was a predictable decrease in Hb concentration throughout hospital stay in both cohorts. This decrease in Hb has been previously shown in other trials in cardiac surgery and is arguably pluri-factorial, due to blood loss, inflammation, and haemodilution [20,22]. Although there was a trend towards a lower proportion of patients requiring PRBC transfusion in the “after” group, this difference was not statistically significant. It is possible that the sample size was not large enough to detect a significant difference, or that other unmeasured factors influenced the results. This finding is atypical in comparison to other results from the literature, where transfusion is reduced after the implementation of PBM measures, despite controversial results regarding other outcomes [23]. This can be explained by a significant barrier to implementation identified in our cohort, which is the short time between the pre-anaesthetic visit and surgery, which was, on average, 1.3 days (SD 1.4) in the “before” group, vs. 0.9 days (SD 0.7) in the “after” group. This is a particularity of the medical system of the country, where there is no systematic ambulatory pre-anaesthetic evaluation with sufficient time before elective surgery. This proves to be a significant barrier to the implementation of preoperative treatment for anaemia, in the absence of a functional pre-hospital care. Furthermore, the worsening during the “after” period is explained by the beginning of the pandemic in 2020 and the associated pressure of minimizing the contact between the patient and the medical system.

Notably, there is a high PRBC transfusion rate in both groups. However, previous studies of large cohorts have reported a large variability in transfusion practice in national and international registries, ranging from 22 to 67% of patients being transfused with at least one unit of PRBC [24,25]. This is not ideal, but these are real-world data from a country where allogeneic blood is not paid for by hospitals, so the incentives to reduce transfusion rates come from scientific societies and practice guidelines, sometimes at odds with hospital administrators, who, at first, pay more for blood conservation measures.

Postoperative Hb was lower in the after group, both in the immediate postoperative period as well as at hospital discharge. This suggests a better compliance of physicians with the rational use of PRBC and adherence to a restrictive transfusion protocol. This approach is supported by several studies in the literature and has been shown to be non-inferior in terms of perioperative outcome in cardiac surgery [26,27,28] and is endorsed by recent guidelines [29].

Although the numerical reduction in PRBC transfusion after matching is not significant, we interpret the results as an encouragement to further add measures in the implemented PBM armamentarium, such as the treatment of anaemia before surgery and the systematic use of cell saver. Future data and regular benchmarking will be necessary to validate this hypothesis.

Fresh-frozen plasma transfusion is a common intervention in cardiac surgery and can lead to significant morbidity, as each unit has a cumulative detrimental effect on perioperative outcomes [30,31]. Therefore, any reduction in FFP transfusion rates is clinically relevant, as it can lead to improved patient outcomes and reduced healthcare costs. The reduction in FFP transfusion rates seen in this study is consistent with previous studies that have reported a decrease in blood product utilization following the implementation of PBM [32]. The decrease in FFP transfusion in the matched cohorts is potentially attributable to more evidence-based transfusion practice, as well as better access to single-factor concentrates (fibrinogen and prothrombin complex concentrates—PCC). This has not been recorded and is a limitation in our study.

PLT was not different between the two groups, but it is to be noted that there is a very low PLT transfusion rate in both groups. This is explainable by the frequent blood product shortage in a system where donation is voluntary, but remains very low, compared to other healthcare systems in Europe [33].

The increase in the utilization of the standardized haemostatic questionnaire is also noteworthy, as it suggests that the implementation of PBM guidelines can lead to changes in clinical practice and improved adherence to evidence-based protocols. The use of standardized questionnaires and checklists has been shown to improve patient safety and reduce medical errors in other areas of anaesthesiology [34]. Our study provides further evidence of their effectiveness in the context of blood management.

The use of a standardized haemostatic questionnaire is independently associated with a reduction in PRBC use in the multivariate regression model. This suggests that, had there been a better uptake of the questionnaire (the implementation was for only 75.7% of patients), it is likely to have led to a reduction in PRBC transfusion in the matched “after” cohort. The result is coherent with the aim of the questionnaire [19], which not only triages patients with a potentially undetected bleeding disorder, but also signals to the physician to adequately adjust or discuss with the multidisciplinary team the management of antiplatelet and anticoagulant agents. In other published data, various risk scores and questionnaires have performed differently in cardiac surgery, so there is no consensus on the best risk assessment score [35].

The positive predictors of transfusion identified in the multivariable analysis confirm our previous study [20].

Age > 64 years was an independent predictor of PRBC transfusion, and this may be explained by the perceived lower tolerance to anaemia of elderly patients, due to expected additional co-morbidities in elderly, frail patients. However, recent data suggest that restrictive transfusion in elderly patients could lead to better outcomes [28].

Female sex was a strong predictor of perioperative PRBC transfusion. This is explained by the smaller blood volume in females and consequently greater haemodilution during CPB. This has been previously reported in other, larger cohorts, where it could have also been linked to the different definition of anaemia between sexes according to the World Health Organization (WHO) [36,37,38].

However, we used a uniform definition of anaemia in this cohort, of Hb < 13 g/dl, irrespective of sex, as recommended by recent consensus papers and expert reviews [39]. Using this definition, preoperative anaemia was still a strong independent predictor of perioperative PRBC transfusion, adding to the data which suggest that the WHO classification might be obsolete.

Fresh-frozen plasma transfusion was independently associated with PRBC transfusion, which seems counterintuitive as our institution does not have a fixed-ration transfusion protocol but rather includes FFP and a staged algorithm as an alternative to PCC in the institutional protocol for the management of acute haemorrhage (Supplementary Figure S3). This association of FFP with PRBC transfusion confirms the previous paper from our group [20], and could be related to the non-compliance with guidelines but also potential residual confounders in our analysis.

This study has several limitations. Firstly, it is a retrospective study which uses observational data, and the possibility of residual confounding despite the use of coarsened exact matching remains. Additionally, the study was conducted in a single centre, which may limit the generalizability of the results. Furthermore, the Romanian PBM recommendations were followed in collecting data, but are not specific to cardiac surgery. Additionally, since the systematic treatment of preoperative anaemia was not yet in place at our institution and was rather anecdotal, data on this aspect were considered not pertinent for our analysis. Finally, most of the patients included in the analysis from the year 2020 were operated on after March 11, when the WHO declared the COVID-19 health crisis as a pandemic, which altered patient management hospital logistics worldwide. This is why, when building the matching model, we included hospital and ICU LOS as potential confounders, and not as outcomes.

## 5. Conclusions

Our before and after study provides further evidence of the effectiveness of PBM l recommendations in reducing blood product utilization and improving adherence to evidence-based protocols in the context of cardiac surgery. Implementing national PBM recommendations was associated with a reduction in FFP transfusion after coarsened exact matching. The adherence to a standardized preoperative bleeding risk questionnaire was independently associated with less PRBC transfusion. Age > 64 years, female sex, FFP transfusion, preoperative anaemia, surgery time, re-exploration for bleeding, ROTEM use, and standardized pretransfusion checklist use were independently associated with PRBC transfusion. Future studies with larger sample sizes and randomized designs are needed to further evaluate the impact of PBM programs on patient outcomes.

## Figures and Tables

**Table 1:** Types of surgeries performed in each analysed period.

*Type of Surgery*	Before (Number and Percentage)	After (Number and Percentage)
*CABG*	252 (35.5%)	171 (36.9%)
*One valve replacement/repair*	256 (36.1%)	144 (31%)
*Two or more valves replacement/repair*	59 (8.3%)	42 (9.1%)
*Valve(s) and CABG*	69 (9.7%)	52 (11.4%)
*Surgery on thoracic aorta*	44 (6.2%)	32 (6.9%)
*Other*	29 (4.1%)	23 (4.9%)

Legend: CABG—coronary artery bypass grafting.

**Table 2:** Perioperative outcomes used as co-variates in the coarsened exact matching model.

*Variable*	Before	After	*p* =	Matched Before	Matched After	*p* =
*Number*	710	464		157	169	
*Age*	64 (58–69)	64 (56–69)	0.643	66 (60–69)	65 (59–69)	0.504
*Sex (M)*	469 (66.1%)	306 (65.9%)	0.970	111 (70.7%)	117 (69.2%)	0.772
*Hospital LOS (days)*	13 (10–16)	14 (11–18)	0.037	12 (9–15)	12 (10–15)	0.412
*ICU LOS (days)*	2 (2–4)	3 (3–4)	<0.001	2 (2–3)	3 (3–3)	<0.001
*Surgical re-exploration* *n (%)*	45 (6.3%)	53 (11.4%)	0.002	0	0	
*Surgery time (mins)*	270 (220–330)	245 (210–290)	<0.001	240 (210–300)	245 (210–280)	0.987
*Hb < 13 g/dl*	288 (40.5%)	183 (39.4%)	0.701	49 (31.1%)	55 (32.5%)	0.796
*Hb1 (g/dl)*	13.4 (12.3–14.4)	13.4 (12.3–14.3)	0.863	13.6 (12.6–14.5)	13.6 (12.7–14.5)	0.844
*Hb2 (g/dl)*	13.3 (12.2–14.4)	13 (11.7–14.1)	<0.001	13.6 (12.6–14.5)	13.2 (12.1–14.2)	0.004
*ROTEM use n (%)*	123 (17.3%)	98 (21.5%)	0.822	6 (3.8%)	7 (4.1%)	0.883
*Cell saver* *n (%)*	7 (1.2%)	7 (1.5%)	0.657	0	0	

Legend: LOS—length of stay, ICU—intensive care unit, Hb—haemoglobin, ROTEM—rotational thromboelastometry.

**Table 3:** Perioperative study outcomes.

*Variable*	Before	After	*p* =	Matched Before	Matched After	*p* =
*PRBC transfusion, n patients (%)*	369 (52%)	266 (57.3%)	0.072	79 (50.3%)	78 (44.9%)	0.334
*Perioperative PRBC transfusion (units, medians, and IQR)*	1 (0–2)	1 (0–3)	0.009	1 (0–2)	0 (0–1)	0.424
*Perioperative PRBC transfusion (units, means, and SD)*	1.39 (2.26)	2.04 (3.88)	<0.001	0.94 (1.32	0.89 (1.32)	0.705
*Intraoperative PRBC transfusion (units, medians, and IQR)*	0 (0–1)	0 (0–1)	0.802	0 (0–1)	0 (0–1)	0.283
*Intraoperative PRBC transfusion (units, means, and SD)*	0.84 (1.46)	0.82 (1.46)	0.797	0.6 (1.01)	0.47 (0.85)	0.190
*Postoperative PRBC transfusion (units, medians, and IQR)*	0 (0–1)	0 (0–1)	0.021	0 (0–1)	0 (0–1)	0.960
*Postoperative PRBC transfusion (units, means, and SD)*	0.67 (1.56)	1.2 (3.4)	<0.001	0.36 (0.64)	0.41 (0.8)	0.529
*Hb3 (g/dl)*	9.9 (8.9–10.8)	9.3 (8.6–10.2)	<0.001	10.1 (9.1–11.1)	9.4 (8.7–10.1)	<0.001
*Hb4 (g/dl)*	9.7 (9–10.6)	9.2 (8.5–9.9)	<0.001	9.8 (9.2–10.7)	9.3 (8.5–9.9)	<0.001
*Perioperative FFP transfusion (units, medians, and IQR)*	0 (0–2)	0 (0–2)	0.348	0 (0–0)	0 (0–0)	0.04
*Perioperative FFP transfusion (units, means, and SD)*	1.02 (2.34)	1.83 (5.27)	<0.001	0.7 (1.73)	0.38 (1.05)	0.04
*Intraoperative FFP transfusion (units, medians, and IQR)*	0 (0–0)	0 (0–0)	0.387	0 (0–0)	0 (0–0)	0.004
*Intraoperative FFP transfusion (units, means, and SD)*	0.76 (1.7)	0.77 (1.92)	0.933	0.59 (1.31)	0.23 (0.79)	0.003
*Postoperative FFP transfusion (units, medians, and IQR)*	0 (0–0)	0 (0–0)	0.002	0 (0–0)	0 (0–0)	0.481
*Postoperative FFP transfusion (units, means, and SD)*	0.29 (1.23)	1.05 (4.38)	<0.001	0.11 (0.73)	0.15 (0.74)	0.591
*PLT transfusion,* *n patients (%)*	65 (9.3%)	40 (8,6%)	0.748	10 (6.3%)	6 (3.5%)	0.239
*Preoperative anaesthetic consultation (days before surgery, median with IQR)*	1 (1–1)	1 (0–1)	<0.001	1 (1–1)	1 (1–1)	0.005
*Preoperative anaesthetic consultation (days before surgery, mean, SD)*	1.32	0.96	<0.001	1.3 (1.4)	0.9 (0.7)	0.002
*Standardised haemostasis questionnaire,* *n patients (%)*	72 (10.14%)	332 (71.8%)	<0.001	19 (12.1%)	128 (75.7%)	<0.001
*Standardized pretransfusion checklist,* *n patients (%)*	0	301 (65.1%)		0	93 (55%)	

Legend: PRBC—packed red blood cells, FFP—fresh-frozen plasma, PLT—platelets.

**Table 4:** Univariate and multivariate analyses of the association between perioperative variables and transfusion risk.

*Variables*	*Univariate*		*Multivariate*	
	OR (95% CI)	*p*	OR (95% CI)	*p*
*Age > 64 years*	2.31 (1.825–2.922)	<0.001	1.652 (1.17–2.331)	0.004
*Female sex*	2.645 (2.048–3.415)	<0.001	2.404 (1.655–3.492)	<0.001
*Hospital LOS (days)*	1.037 (1.021–1.054)	<0.001	1.02 (0.994–1.047)	0.138
*ICU LOS (days)*	1.168 (1.097–1.243)	<0.001	1.065 (0.995–1.14)	0.067
*Surgery time (hours)*	1.348 (1.219–1.489)	<0.001	1.295 (1.126–1.488)	<0.001
*Hb < 13 g/dl*	4.315 (3.34–5.576)	<0.001	3.611 (2.528–5.158)	<0.001
*Re-exploration for bleeding*	6.879 (3.717–12.733)	<0.001	3.988 (1.248–12.738)	0.020
*ROTEM use*	4.401 (3.053–6.344)	<0.001	2.18 (1.34–3.544)	<0.001
*FFP transfusion*	7.898 (5.601–11.135)	<0.001	4.023 (2.426–6.671)	<0.001
*PLT transfusion*	3.32 (2.059–5.354)	<0.001	0.951 (0.478–1.892)	0.887
*Standardised haemostasis questionnaire use*	0.766 (0.6–0.976)	0.031	0.565 (0.371–0.861)	0.008
*Standardized pretransfusion checklist use*	8.151 (5.73–11.596)	<0.001	8.875 (5.496–14.332)	<0.001

## Data Availability

Restrictions apply to the availability of these data. The data presented in this study are available on reasonable request from the corresponding author.

## Supplementary Materials

The following supporting information can be downloaded at: https://www.mdpi.com/article/10.3390/jcdd10070266/s1, Figure S1: Standardized pre-operative haemostasis questionnaire, Figure S2: Standardized pre-transfusion checklist, Figure S3: Bleeding management standardized procedure, Table S1: PBM report form.
