# Supplementary material for: Less (Transfusion) Is More—Enhancing Recovery through Implementation of Patient Blood Management in Cardiac Surgery: A Retrospective, Single-Centre Study of 1174 Patients
Source: J Cardiovasc Dev Dis. 2023 Jun 22;10(7):266. doi: 10.3390/jcdd10070266 (PMC10380242; doi:10.3390/jcdd10070266)

BLEEDING MANAGEMENT

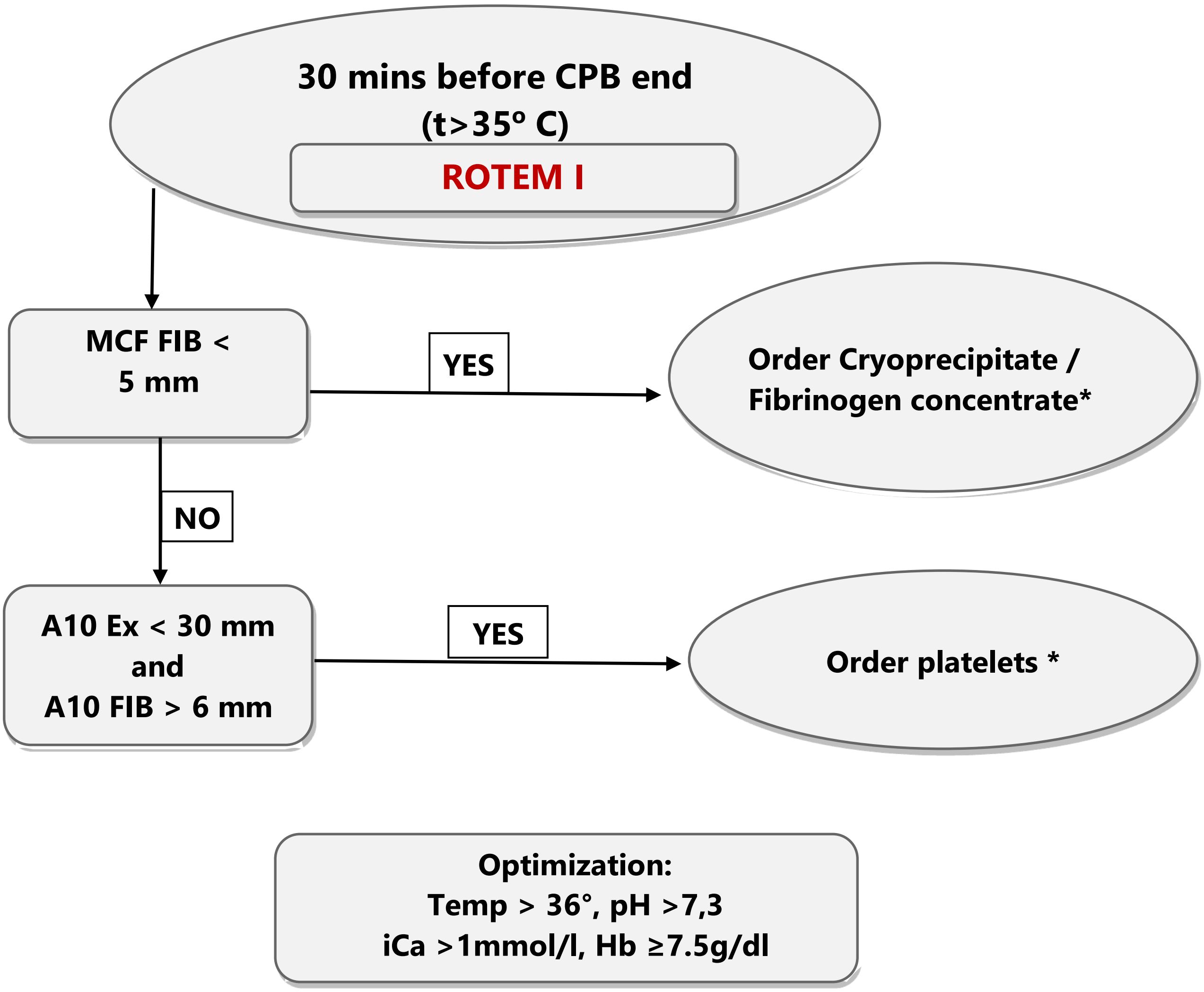

*Can be administered with protamine if massive bleeding in acute aortic dissection surgery

IF THE PATIENT IS NOT BLEEDING THE SECOND ROTEM IS NOT MANDATORY

ROTEM II

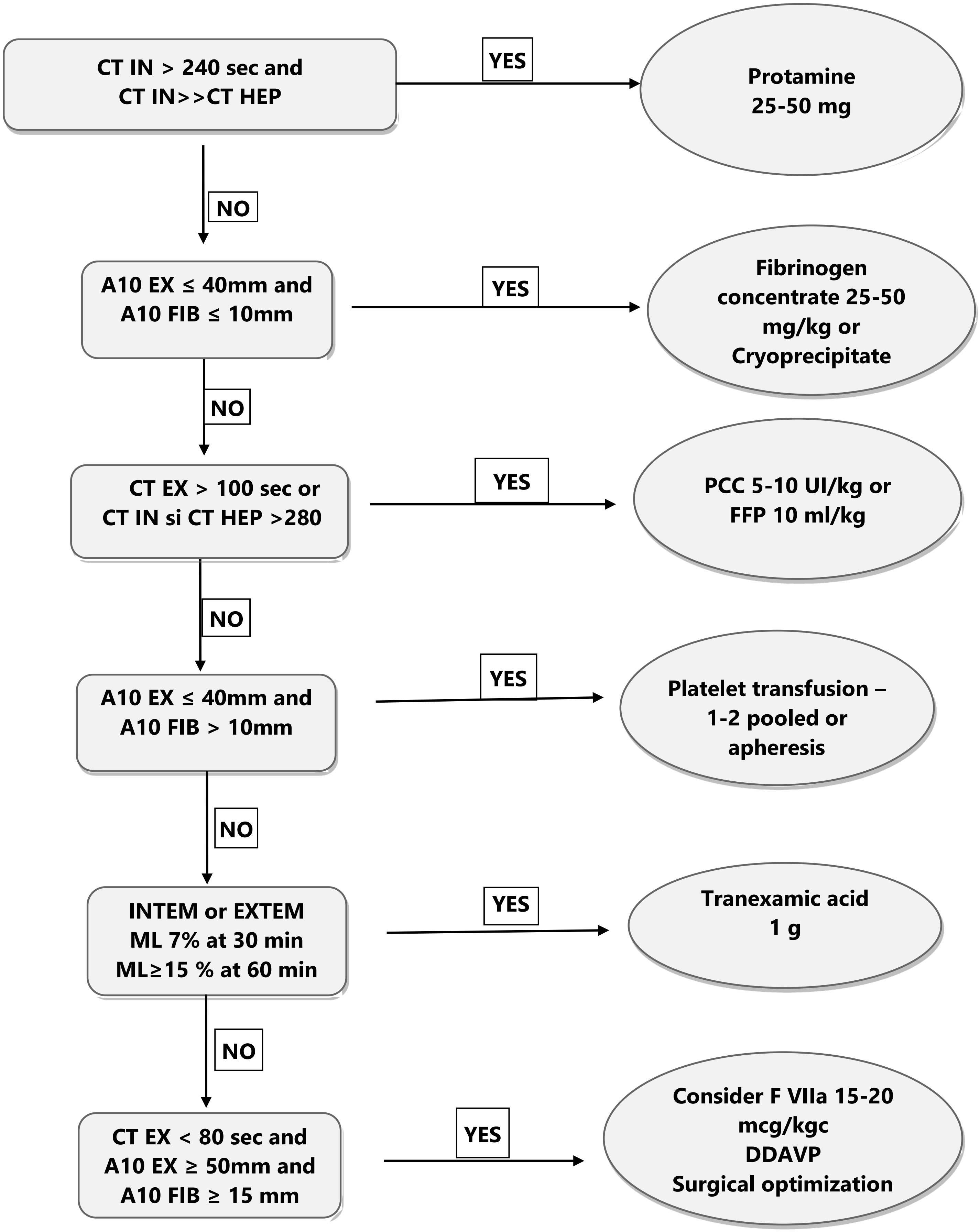

Supplement: Supplementary file 1 [file jcdd-10-00266-s001.zip › Supplementary figure 3.pdf]
