# Supplementary material for: Less (Transfusion) Is More—Enhancing Recovery through Implementation of Patient Blood Management in Cardiac Surgery: A Retrospective, Single-Centre Study of 1174 Patients
Source: J Cardiovasc Dev Dis. 2023 Jun 22;10(7):266. doi: 10.3390/jcdd10070266 (PMC10380242; doi:10.3390/jcdd10070266)
Supplement: Supplementary file 1 [file jcdd-10-00266-s001.zip › Supplementary table 1.pdf]

APPENDIX 5. Patient Blood Management Programme Monitoring and Evaluation Benchmarks

COC*		Date of admission		Age		Gender (M/F)	

Diagnosis:<br>1.<br>2.<br>3.

**Preoperative evaluation date:**				**Number of days between preoperative evaluation and surgery:**				
**Lab investigations – tests (as per Protocol algorithm):**								**(Absolute/ Functional) Iron deficiency (Y/N)**
Hb (g/dL)		TSAT (%)		Serum ferritin (ng/mL)		CRP (mg/L)		
**Iron deficiency treatment:**								
IV iron administration (Y/N)		IV iron dose (mg)		ESA** administration (Y/N)		ESA dose		
Days before surgery of iron administration		Rescue erythropoiesis (Y/N)						
**Haemostasis:**								
Positive answer to haemostasis questionnaire (Y/N):				Haemostasis disorder detected (Y/N):				
Adjustment of antiplatelet drug therapy (Y/N):								
Point of care haemostasis tests (Y/N):				Haemostatic drugs:				

Surgery:

Type of surgery		
Date of surgery		

Length of stay in hospital (days):		Length of stay in ICU (days):		In-hospital death (Y/N)	

Haemoglobin (Hb, g/dL):					
At first preoperative evaluation:		Preoperative:		Postoperative:	
At discharge					

Blood loss minimisation:					
Use of micro tubes for specimen collection (Y/N):		Use of closed flush systems for arterial lines and central venous catheters (Y/N):		Use of blood salvage systems (Y/N):	

Acute haemorrhage (Y/N):		Use of the multidisciplinary protocol for acute haemorrhage (Y/N):	
Optimisation of the patient's tolerance of anaemia:			
Use of advanced haemodynamic monitoring (Y/N):			
Use of hyperoxia (Y/N):		Monitoring of anaesthetic depth or neuromuscular block (Y/N):	

Transfusions (number of units for each component):						
Packed red blood cells	Preoperative:		Intraoperative:		Postoperative:	
Platelet concentrate	Preoperative:		Intraoperative:		Postoperative:	
Fresh frozen plasma	Preoperative:		Intraoperative:		Postoperative:	
Other blood component: ____________________	Preoperative:		Intraoperative:		Postoperative:	

Indication code for transfusion (see Appendix 4)	

Postoperative complications	YES	NO	
General postoperative complications - during hospital stay (Y/N), see Glossary:			Comments
Acute kidney injury			Stage:
Major adverse cardiac events (MACE)			
Myocardial infarction			
Pulmonary embolism			
Pneumonia			
Postoperative haemorrhage			
Stroke			
Surgical site infection (deep)			
Surgical site infection (superficial)			
Other complications (based on the type of surgery):	YES	NO	Comments
-<br>-			
Transfusion-related complications			
