# Supplementary material for: Less (Transfusion) Is More—Enhancing Recovery through Implementation of Patient Blood Management in Cardiac Surgery: A Retrospective, Single-Centre Study of 1174 Patients
Source: J Cardiovasc Dev Dis. 2023 Jun 22;10(7):266. doi: 10.3390/jcdd10070266 (PMC10380242; doi:10.3390/jcdd10070266)
Supplement: Supplementary file 1 [file jcdd-10-00266-s001.zip › Supplementary figure 1.pdf]

Supplementary figure 1 - Standardized pre-operative haemostasis questionnaire

**Please tick or fill in the answer (Yes or No):**		**No**	**Yes**	**Validation**
Have you ever been diagnosed with a kidney, liver or bone marrow disease?				
Have you ever noticed any of the following types of bleeds, sometimes without an apparent cause?				
a)	Nosebleeds (without other causes like prolonged sneezing, dry air, blowing your nose too hard, etc.)			
b)	Large bruises or bleeding spots (also on the trunk, occurring even in the absence of trauma/hits); provide diameter.			
c)	Bleeding joints, soft tissue or muscles			
Have you ever had prolonged bleeding in case of cuts or scratches?				
Do you remember having massive or prolonged bleeding after tooth extractions?				
Do you remember having prolonged bleeding during or after surgery?				
Do your wounds take a lot of time to heal?				
Have you ever had cases of haemophilia or bleeding disorders in your family?				
Do you take or have you taken blood thinners lately? (e.g. Sintrom, Trombostop, Pradaxa, Eliquis, Xarelto, Aspenter, Plavix, Brilique, Efient, etc.)				
Are you currently on pain killers or anti-rheumatics, even over-the-counter ones? (e.g. Aspirin, Voltaren, Ibuprofen, etc.)				
Have you ever had an abnormal coagulation test or anaemia?				
**Additional questions for women:**				
Do you have a long (>7 days) and/or heavier (frequent tampon/pad change) period?				
Have you had massive bleeding after childbirth or during your period?				
