# Supplementary material for: Less (Transfusion) Is More—Enhancing Recovery through Implementation of Patient Blood Management in Cardiac Surgery: A Retrospective, Single-Centre Study of 1174 Patients
Source: J Cardiovasc Dev Dis. 2023 Jun 22;10(7):266. doi: 10.3390/jcdd10070266 (PMC10380242; doi:10.3390/jcdd10070266)
Supplement: Supplementary file 1 [file jcdd-10-00266-s001.zip › Supplementary figure 2.pdf]

Supplementary figure 2 – standardized pre-transfusion checklist

Hb <6g/dL

a. □ Irrespective of compensatory capacity

Hb 6-8g/dL

a. □ Indicators of anaemic hypoxia (tachycardia, hypotension, ischemia on ECG, lactic acidosis)

b. □ Reduced compensatory capacity, presence of risk factors (coronary disease, heart failure, cerebrovascular diseases)

c. □ Other indications...

Hb 8-10g/dL

a □ Indicators of anaemic hypoxia (tachycardia, hypotension, ECG ischemia, lactic acidosis)

b. □ Other indications ....

Hb >10 g/dL

a. □ Other indications ....
